# pH-responsive shell-sheddable polymeric nanoparticles as the photodynamic drug carrier: synthesis, characterization, and in vitro studies

**DOI:** 10.55730/1300-0152.2643

**Published:** 2022-12-15

**Authors:** JING LU

**Affiliations:** Division of General Education, Seokyeong University, Republic of Korea

**Keywords:** pH-responsive nanoparticles, protophorphyrin IX, sheddable nanocarriers, phototoxicity

## Abstract

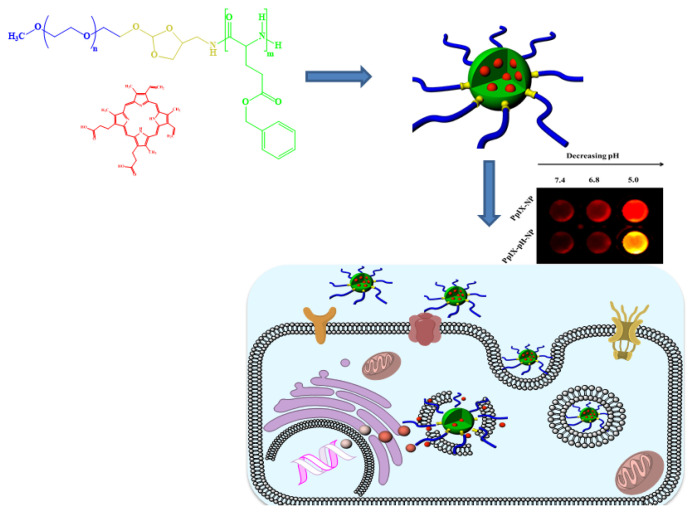

Shell-sheddable nanoparticles, composed of amphiphilic blockpolymers, have emerged as an attractive vehicle for the site-specific delivery of therapeutic agents. In this study, pH-responsive sheddable copolymers bearing an orthoester linker were synthesized via the ring-opening polymerization between γ-benzyl-L-glutamate N-carboxyanhydride and orthoester-bearing poly (ethylene glycol) macroinitiator (PEG-pH-NH_2_). The obtained poly (ethylene glycol)-*b*-poly(γ-benzyl-L-glutamate) (PEG-PBLG) could form stable nanoparticles in aqueous solutions due to the amphiphilic nature of the block copolymers. The PEG-PBLG–based nanoparticle exhibited good stability in physiological conditions (pH 7.4), whereas the nanoparticle was disassembled under acidic conditions (pH 5.0). The nanoparticles could encapsulate a photosensitizer, protophorphyrin IX (PpIX), and deliver it into acidic environments. According to optical imaging test, it was found that quenched fluorescence signal of PpIX highly recovered under acidic conditions. Acid-responsive sheddable nanoparticles rapidly release the PpIX when they are incubated under acidic conditions (pH 5.0), and the PpIX release was remarkably reduced in physiological buffer (pH 7.4). In vitro cytotoxicity test showed that cells treated with pH-responsive sheddable nanoparticle became highly phototoxic upon irradiation. Microscopic observation demonstrated that PpIX-loaded nanoparticle rapidly degraded at the endosome of SCC7 cancer cells, which enabled PpIX release into the cancer cells. These results suggest that pH-responsive sheddable are a promising carrier for photodynamic agents.

## 1. Introduction

Cancer is the one of the leading causes of death worldwide (Sung et al., 2021). Various therapies such as photodynamic therapy (PDT), chemotherapy, immunotherapy, and radiotherapy are available for the treatment of cancer. In PDT, the photosensitizers generate singlet oxygen or reactive oxygen species (ROS) which can destroy the cells upon irradiation. However, it has limitations such as normal cell toxicity, reduced bioavailability, nontargeted delivery, and inability to overcome biological barriers. In order to overcome the limitations of conventional therapies, nanoparticles with modifications have gained great attention for treatment specificity such as precision applications (Mitchell et al., 2021). Among them, smart polymeric nanoparticles self-assembled from biodegradable amphiphilic copolymers with stimuli-responsive linkers have considered promising delivery carriers for the triggered release of bioactive agents (Lee et al., 2009; Wang et al., 2021). The distinct physicochemical characteristics of nanoparticles, including thermodynamic stability, biocompatibility, and high amount of bioactive agent imbibing capacity, have received great attention in drug delivery. In particular, systemically administered nanoparticles can effectively circulate in the blood without being detected by the immune system. Upon reaching tumor tissues, the nanoparticles effectively escape through the leaky vessels and this phenomena is called the enhanced permeation and retention (EPR) effect, which can increase the deposition of nanoparticles into the tumor regions (Gagliardi et al., 2021). However, there is a serious limitation in effective cancer therapy using conventional nanoparticles in EPR-based drug release system.

In general, conventional nanoparticles release the drug in a sustained manner, and the amount of drug release from the nanoparticles is insufficient for effective eradication of tumor. The anticancer activities of the nanoparticles are often enhanced by triggering the drug release. Recently, polymeric nanoparticles that specifically release the drug in response to the intracellular stimuli such as mildly acidic pH, temperature, hypoxia, and enzymes (Lee et al., 2009; Lee et al., 2011; Chang et al., 2021; Wang et al., 2021) (Thambi et al., 2014; Deirram et al., 2019; Phan et al., 2022), have been paid great attention in drug delivery. These nanoparticles effectively circulate in the bloodstream and allows passive accumulation of nanoparticles to tumors tissues (Han et al., 2015). In order to achieve better circulation of nanoparticles in the bloodstream, hydrophilic ‘stealth’ polymers such as poly (ethylene glycol) (PEG), dextran, or hyaluronic acid are used to fabricate nanoparticles (Thambi et al., 2016; Thambi et al., 2017). These hydrophilic polymers not only enhance the circulation of nanoparticles in vivo but also safeguard nanoparticles from proteolytic digestion and aggregation with blood proteins (Manivasagan et al., 2022). Meanwhile, upon reaching target site of interest, nanoparticles disassemble and trigger the release of encapsulated bioactive agents for better therapeutic response. For this purpose, various responsive linkers such as disulfide linkers (-S-S-) (Thambi et al., 2011), reactive oxygen species (ROS) (-Se-Se- or thioketal linkers) linkers, hypoxia-responsive linkers (-N=N-), and enzyme-responsive peptides are introduced between hydrophilic and hydrophobic polymers (Thambi and Park, 2014). In addition, pH-responsive linkers such as ketal-based linkers that exhibit the scission of nanoparticle as hydrophilic and hydrophobic segments are used (Lee et al., 2011). Some charge switchable polymers exhibit charge transition from neutral to positive or from negative to positive when the pH is altered (Li et al., 2018). These polymers such as poly (histidine), poly (β-aminoester), poly (4-vinyl pyridine), and poly (diisopropylamino ethyl methacrylate) possess tertiary amine groups (Li et al., 2019). Therefore, introduction of pH-responsive polymers or pH-responsive linkers in the nanoparticles undergo phase transition from hydrophobic/amphiphilic to hydrophilic as a function of change in environmental pH. It is well known that pH-responsive polymers such as poly (β-amino ester), poly (N,N′-dimethyl aminoethyl methacrylate), and poly (2-(diisopropylamino) ethyl methacrylate)-based polymers exhibited sharp pH-sensitive micellization/de-micellization transition or disassembly of the nanostructure and the pH-sensitivity of these polymers are controlled by varying molecular weight and block length (Min et al., 2010; Ma et al., 2014; Tang et al., 2019). These polymers are used to prepare polymeric nanoparticles for loading chemotherapeutic agents such as doxorubicin, paclitaxel, and camptothecin. At the physiological condition, these polymers can effectively imbibe drugs into the hydrophobic core of the nanoparticles. When these nanoparticles are exposed to acidic pH condition or reached periphery of the tumors by the tumor targeting or passive targeting, rapid disassembly of the nanostructures triggers the release of anticancer drugs specifically at the tumor tissues. On the other hand, minimal drug release was observed in the systemic circulation that minimizes the side effects.

**Figure 1:**
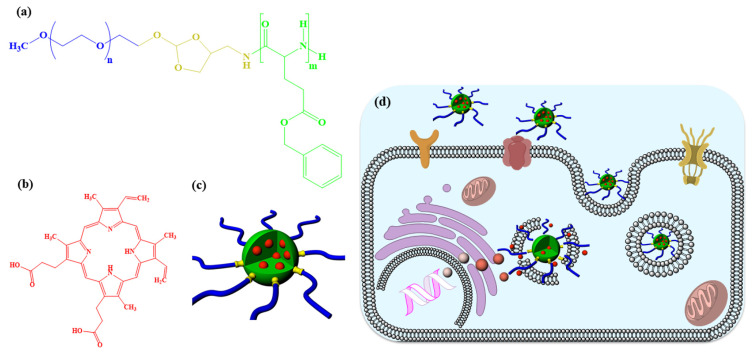
Chemical structure of (a) PEG-PBLG copolymers and (b) PpIX. (c) Schematic illustration of (c) pH-NPs in aqueous condition. (d) Internalization of PpIX-NPs into the cancer cells, subsequent release of PpIX at the intracellular environment. Irradiation of PpIX using NIR laser allowed the generation of singlet oxygen and induced apoptosis.

In this study, pH-responsive shell sheddable nanoparticles were used for the encapsulation and delivery of water-insoluble photosensitizer protophorphyrin IX (PpIX) (Figure 1). The shell-sheddable amphiphilic copolymer was prepared via ring-opening polymerization of benzylglutamate N-carboxy anhydride (NCA) using pH-responsive orthoester containing PEG macroinitiator. The PpIX was loaded into the nanoparticles using solvent casting technique. The advantage of pH-responsive shell sheddable nanoparticles in PpIX delivery was examined by various systematic studies including stability, drug release, and in vitro phototoxicity studies at different pH values (pH 5.0 and pH 7.4).

## 2. Experimental procedures

### 2.1. Materials

PEG-NH_2_ (M_n_ = 5000 g mol^−1^) was obtained from Laysan Bio Inc. (Arab, AL, USA). Benzyl glutamate (BLG), 3-amino-1,2-propanediol, trifluoroethyl acetate, PEG-NH_2_ (M_n_ = 5000 g mol^−1^), triphosgene, 4,6-diamino-2-phenylinodole (DAPI), and protoporphyrin IX (PpIX) were obtained from Sigma-Aldrich Co. (St. Louis, MO, USA).

### 2.2. Preparation of pH-responsive nanoparticles

The pH-responsive copolymers were synthesized as previously reported (Thambi et al., 2011) . In brief, BLG NCA (0.72 g, 2.74 mmol) in DMF was added into PEG-pH-NH_2_ (1 g, 0.196 mmol) in DMF and stirred at 35 °C for 24 h. The resultant PEG-pH-PBLG copolymer was precipitated in diethyl ether, filtered, and dried. Similarly, the control PEG-*b*-PBLG copolymer without the orthoester-linker was synthesized using mPEG-NH_2_ macroinitiator.

In order to prepare the pH-responsive nanoparticles, 100 mg of PEG-pH-PBLG copolymers was dissolved in DMSO (5 mL), and stirred vigorously for 1 h to obtain a clear solution. Thereafter, the clear solution was transferred to cellulose membrane (MWCO: 3,500 Da) and dialyzed against distilled water 24 h. Finally, the dialysate was passed through 0.80 μm filter and lyophilized to obtain pH-responsive nanoparticles.

### 2.3. Characterization

The structure and composition of the PEG-PBLG copolymers were analyzed using ^1^H NMR spectra (JNM-AL300, JEOL, Tokyo, Japan). The particle size of PEG-PBLG copolymer was measured using a FPAR-1000 particle analyzer (Otsuka Electronics, Osaka, Japan). The stability of the nanoparticles was measured in pH 7.4 and pH 5.0 along with time. The morphology of the nanoparticles was investigated using FE-TEM (JEM-2100F, JEOL, Tokyo, Japan) imaging.

### 2.4. PpIX-loaded pH-responsive nanoparticles (PpIX-pH-NPs) preparation

PpIX-loaded nanoparticles were fabricated by dialysis method (Lee et al., 2009). Briefly, the PEG-pH-PBLG copolymer was dissolved in DMSO. PpIX dissolved in DMSO was mixed into the copolymer solution, and vigorously stirred. The reaction mixture was then poured into the dialysis membrane tube (MWCO = 3500 Da) and dialyzed using excess water for 1 day. Thereafter, the solution was passed through a 0.8-μm filter before being lyophilized. An identical method was applied to prepare control PpIX-loaded nanoparticles (PpIX-NPs).

The loading efficiency and content of PpIX in the nanoparticles were calculated using UV-vis spectrophotometer by following a previously reported procedure (Yu et al., 2019).

In order to quantify the amount of PpIX in the nanoparticles, PpIX-loaded nanoparticles were dissolved using organic reagent with ultrasonication and the concentration of PpIX in the solution was measured using UV-vis spectrophotometer at 370 nm. The loading efficiency (LE) and loading content (LC) were calculated using the following formula:


$$\begin{array}{l} \text{LE }(\%)=\text{Wt. of PpIX in the nanoparticles}/\text{Wt. of PpIX in feed}\\ \text{LC }(\%)=\text{Wt. of PpIX in the nanoparticles}/\text{Wt. of nanoparticles.} \end{array}$$

### 2.5. In vitro PpIX release and stability of nanoparticles

To investigate release pattern of PpIX from PpIX-pH-NPs and PpIX-NPs (3 mg), the lyophilized nanoparticles were suspended in different buffers (pH 7.4 and pH 5.0) and vortexed for 30 s. Thereafter, the dispersion medium was moved to dialysis membrane (MWCO: 3,500) and immersed into 50-mL falcon tubes containing 30 mL of buffer. The tube was gently shaken in a water bath at 37 °C with 100 rpm. At different time points, release medium was withdrawn and replaced with fresh medium. The release sample was analyzed using UV-vis spectrometer to measure the amount of PpIX in the medium.

To quantify the release of PpIX from the nanoparticles, the release medium withdrawn at different time points were subjected to UV-vis spectra analysis. In brief, the concentration of PpIX was analyzed by measuring the absorbance of PpIX at 370 nm by using a UV-vis spectrophotometer and comparing with the standard curve of PpIX (Yu et al., 2019).

To investigate the stability of the nanoparticles, PEG-pH-PBLG and PEG-PBLG copolymers were dispersed in different buffers (pH 7.4 and pH 5.0) and the size of the nanoparticles were measured as a function of time.

### 2.6. In vitro release imaging using optimal imaging test

To further confirm the release of PpIX, PpIX-loaded nanoparticles were dispersed into different pHs (pH 7.4, pH 6.8, and pH 4.5) and transferred to flat-bottomed plates. After 30 min of incubation, the release of PpIX in the well was imaged using a Kodak Image Station 4000MM containing a 12-bit CCD camera (New Haven, CT, USA).

### 2.7. In vitro cell studies

#### 2.7.1. Cell culture

SCC7 cells were cultured in RPMI 1640 medium with 10% (v/v) fetal bovine serum and 1% (w/v) penicillin-streptomycin at 37 °C in a humidified atmosphere. After 48 h, the cells were trypsinized and seeded in a 96-well plates (cell density = 1 ‘ 10^4^ cells/well).

#### 2.7.2. Intracellular PpIX release

PpIX release from nanoparticles was observed using fluorescence quenching and dequenching technique (Lee et al., 2009). For the release test, PpIX-pH-NPs and PpIX-NPs were incubated with SCC7 cells for 12 h. SCC7 cells then were washed with pH 7.4 PBS and fixed using 4% formaldehyde solution followed by staining the nucleus using DAPI. The intracellular localization of PpIX was observed using a microscope (Olympus, Tokyo, Japan).

#### 2.7.3. In vitro anticancer effect and phtotoxicity test

To observe biocompatibility and anticancer activity, the cancer cells treated with nanoparticles containing 1, 2.5, 5, 10, 12.5 μg/mL of PpIX. The cancer cells incubated with culture media were used as a control. After 24 h, the cells were washed to remove free polymer and incubated with fresh culture medium. The 20 μL of 3-(4,5-dimethylthiazol-2-yl)-2,5-diphenyltetrazolium bromide (MTT) solution at 5 mg/mL in PBS was added to each well and incubated for 4 h at 37 °C. Finally, the culture medium was discarded and dissolved in DMSO, and the absorbance of formazan crystals at 570 nm was measured using a microplate reader.

For the phototoxicity test, the cells were irradiated with He–Ne laser (633 nm, 3 mW/cm^2^) for 10 min right after incubating the PpIX-loaded nanoparticles.

## 3. Results and discussion

### 3.1. Synthesis of PEG-pH-PBLG block copolymers

**Figure 2:**
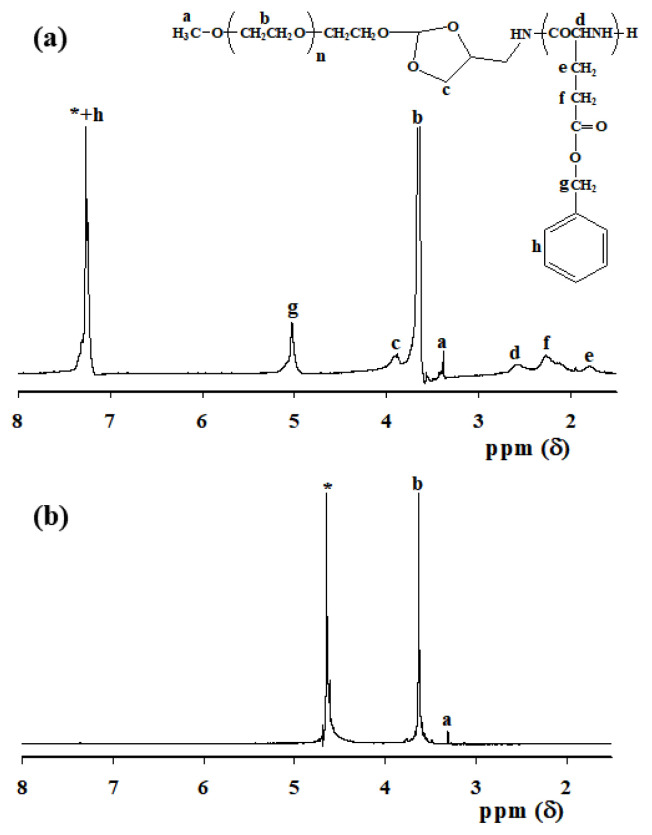
^1^H NMR spectra of PEG-PBLG copolymers in (a) CDCl_3_ and (b) D_2_O.

The PEG-pH-PBLG block copolymer was prepared by ring-opening polymerization of BLG-NCA using PEG-pH-NH_2_ macroinitiator. ^1^H NMR spectra in Figure 2a shows the characteristics peaks of PEG-pH-PBLG block copolymers and the characteristics peaks are well assigned to the copolymer. The polymerization degree of BLG units was also calculated from ^1^H NMR spectra by comparing the integral values of methylene protons of BLG at 5.06 ppm with characteristics methylene proton of PEG at 3.66 ppm. From the ^1^H NMR spectrum, it was found that the polymerization degree of BLG was 12 and the number average molecular weight was found to be 7560 g/mol (Table).

Furthermore, the self-assembly of PEG-pH-PBLG block copolymer was also investigated using ^1^H NMR spectra (Figure 2b). Unlike chlorinated solvents (e.g., CDCl_3_) that dissolve both PEG and PBLG, D_2_O can only dissolve PEG but not hydrophobic PBLG. As expected, the ^1^H NMR spectra of PEG-pH-PBLG block copolymers recorded in D_2_O shows only the characteristics PEG peak. This result suggested that upon mixing with D_2_O, only PEG peaks are solvated and hydrophobic nature PBLG minimizes its contact with water and form a hydrophobic core covered by PEG shell, which is consistent with previously reported amphiphilic copolymer-based nanoparticles (Thambi et al., 2013).

### 3.2. Preparation of and characterization of nanoparticles

**Figure 3:**
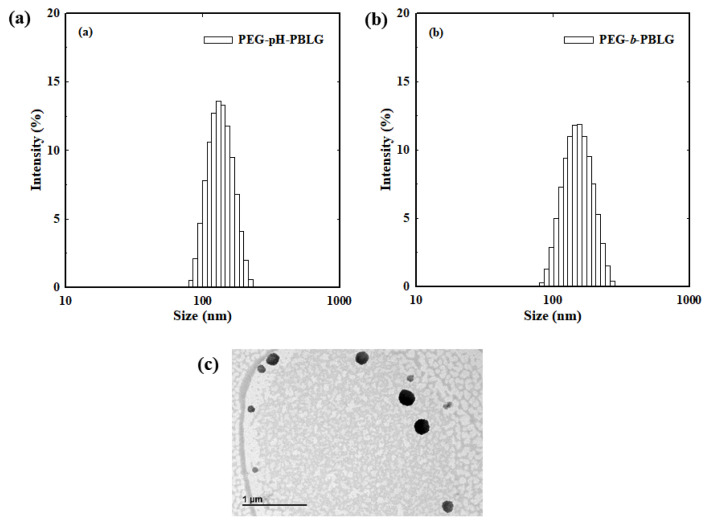
Size distribution of (a) pH-NPs and (b) control NPs in aqueous conditions. (c) TEM image of copolymers formed spherical nanoparticles.

The systematic design of PEG-pH-PBLG block copolymers can produce micelle-like nanoparticles due to the presence of optimal hydrophilic and hydrophobic balance. In aqueous solutions, rationally designed PEG-pH-PBLG copolymers self-assembled into core-shell structure with well-defined hydrophilic PEG shell for prolonged circulation in the blood stream and PBLG hydrophobic core for loading chemotherapeutic drugs and imaging agents. Nanoparticles were fabricated by simple sonication of the PEG-pH-PBLG block copolymers in aqueous solutions. From the DLS study, it was found that PEG-pH-PBLG block copolymers formed a stable nanoparticle with a size of approximately 130 nm (Figure 3a). The control PEG-*b*-PBLG copolymers without pH-sensitive linkers also formed nanoparticles with a size of approximately 145 nm (Figure 3b). Size distribution results showed that both PEG-pH-PBLG and PEG-*b*-PBLG block copolymers formed stable nanoparticles with narrow size distribution, which implied that both copolymers are homogenously dispersed in aqueous solutions. In addition, Figure 3c shows a TEM image of spherical nanoparticles formed by the copolymers.

### 3.3. Stability of nanoparticles in vitro

**Figure 4:**
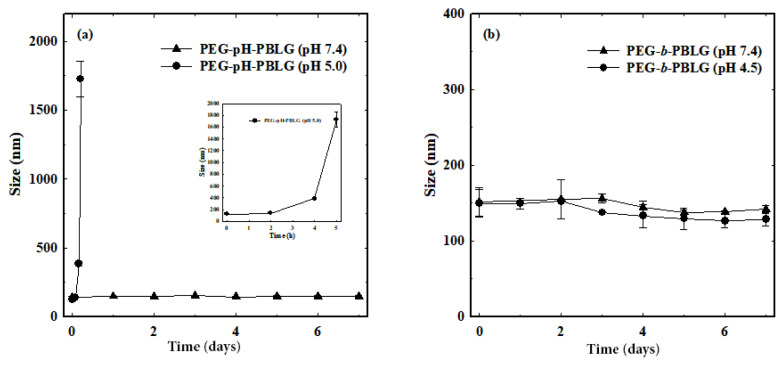
Stability of (a) pH-NPs and (b) control NPs at different pH conditions.

**Figure 5:**
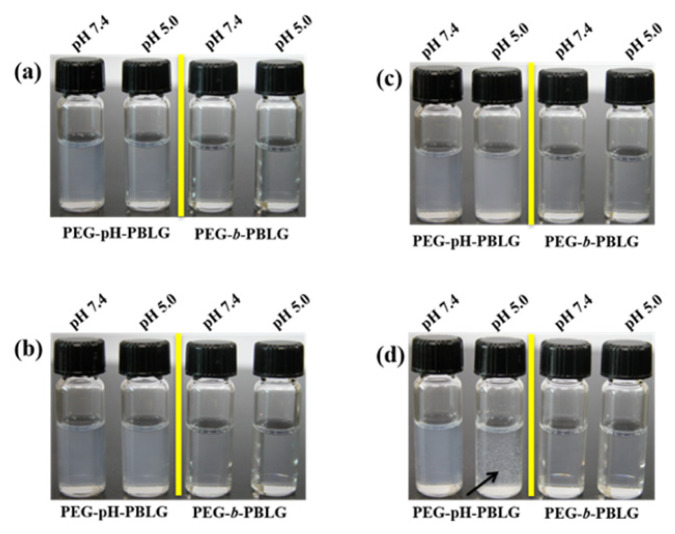
Stability photographs of pH-NPs and control NPs incubated in different pH conditions (pH 7.4 and pH 5.0) at different time points (n = 3). Photographs were taken at (a) 0 h, (b) 2 h, (c) 3 h, and (d) 6 h time intervals.

The stability of polymeric nanoparticles in the buffer conditions is more important for their preferential accumulation in target site. Nanoparticles with poor stability limits drug loading capability or imbibing capacity of chemotherapeutic drug (Thambi et al., 2014). In general, nanoparticles should be well dispersed in physiological conditions and maintain its stability for a long time, whereas, upon reaching the target site, the nanoparticles should exhibit phase transition and disassemble the core-shell structure, which allows the release of loading therapeutic or imaging agents (Sun et al., 2010). In this study, the stability of synthesized nanoparticles (NPs and pH-NPs) was examined by incubating them with PBS buffers (pH 5.0 and pH 7.4) and their size changes were measured as a function of time (Figure 4). The nanoparticles incubated under pH 7.4 exhibited no change in the particle size over 1 week, indicating the stability of the nanoparticles in physiological conditions. Interestingly, when the nanoparticles were incubated under endosomal/lysosomal acidic pH conditions (pH 5.0), the pH-NPs exhibited significant size increase from 145 nm to 1751 nm. At first, the pH-NPs incubated under acidic pH condition showed transparent and when the time progressed, it started forming clouds and started precipitating at 6 h (Figure 5). This is due to the shedding of the PEG shell, resulting disassembly of the core-shell structure led to the precipitation of hydrophobic PBLG. It should be noted that the control NPs exhibit no change in both pH conditions. These results indicated that the presence of pH-responsive orthoester linker present in the pH-NPs induced the disassembly of the NPs at acidic conditions. In contrast, pH-NPs maintained stability under the physiological condition. From the stability test, it is evident that the pH-NPs can maintain their stability during circulation in the blood stream or maintains their stability upon incubation in cell culture medium. Nanoparticles can be decomposed after internalization into cells or exposure to tumor tissues. Therefore, drug loaded pH-NPs may release the encapsulants specifically at the acidic pH conditions.

### 3.4. In vitro release test

**Figure 6:**
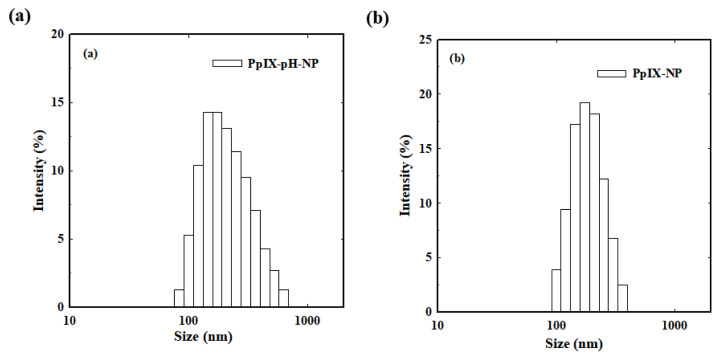
Size distribution of (a) PpIX-pH-NPs and (b) control PpIX-NPs in aqueous conditions.

In order to assess the loading capacity of nanoparticles, PpIX, a water-insoluble photosensitizer, was used. The PpIX was loaded into the nanoparticles by dialysis method. In aqueous condition, the hydrophilic PEG shell tends to be outside and hydrophobic PpIX is located in the inner hydrophobic PBLG core by combination of hydrophobic and π-π interactions. The loading efficiency of PpIX was greater than 95% when the feed ratio was 5 wt.%, which indicates that the nanoparticle system based on PEG-PBLG has good loading capacity. The PpIX-loaded nanoparticles exhibited good dispersion and the size was increased from 130 nm to 208 nm, indicating that effective loading of the drug within the hydrophobic core (Figure 6).

**Figure 7:**
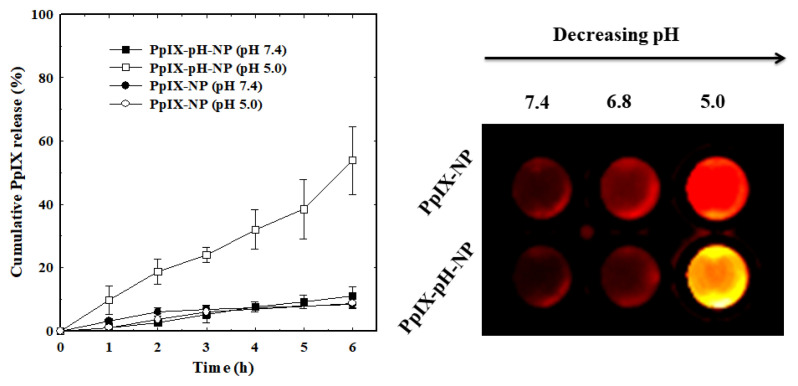
In vitro release of PpIX from nanoparticles under different pH conditions (n = 3).

Accordingly, in vitro release characteristics of PpIX-loaded nanoparticles were measured in different pH conditions to examine the pH-sensitivity of the nanoparticles (Figure 7a). At the physiological condition (pH 7.4), both nanoparticles effectively reduced the initial burst release and showed less than 10% drug release for at least 6 h. Interestingly, the drug release was triggered for PpIX-pH-NPs when they were incubated under acidic pH condition (pH 5.0). The drug release was gradually increased and 53% of PpIX was released from nanoparticles within 6 h. At the same condition, the release pattern of PpIX from control NPs was similar to the nanoparticles incubated in pH 7.4. Such distinct and specific release of PpIX from PpIX-pH-NPs is mainly due to the presence of orthoester linker in the middle of the core-shell nanoparticles. The PEG-pH-PBLG copolymers prepared in this study could self-assemble into nanoparticles at the physiological condition. During the self-assembly process, PpIX is loaded into the hydrophobic core of the nanoparticles. At acidic pH condition (pH 5.0 and pH 6.8), the pH-sensitive linker present at the core-shell interface is hydrolyzed. At first, hydrolysis at the interface leads to the swelling of the nanoparticles that lead to the leakage of the loaded PpIX. Subsequently, the nanoparticle is disassembled and triggers the PpIX release. This is consistent with the previous report based on disassembly of PEG-SS-PCL and Dex-SS-PCL nanoparticles (Sun et al., 2009; Sun et al., 2010).

The pH-sensitivity of PpIX-pH-NPs was further examined by fluorescence quenching and dequenching test (Figure 7b). At the physiological condition, the fluorescence of PpIX was quenched in the hydrophobic core of nanoparticles by self-quenching. On the other hand, the fluorescence of PpIX was recovered under acidic condition, indicating the pH-sensitivity of the nanoparticles. As expected, the fluorescence was not recovered in control nanoparticles, indicating the role of conditions and specifically release PpIX at the endosomal pH-sensitivity in PpIX release. acidic conditions.

### 3.5. Cellular uptake of nanoparticles

**Figure 8:**
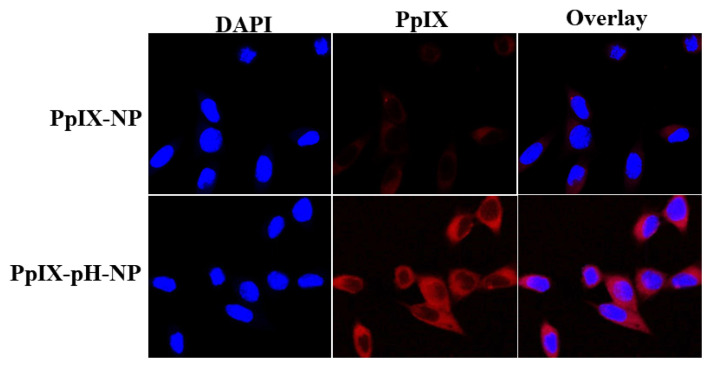
Cellular uptake of PpIX-pH-NPs and PpIX-NPs in SCC7 cells.

To confirm the effective internalization and release of PpIX from nanoparticles, cellular uptake of SCC7 cells were examined using fluorescence imaging after exposure with nanoparticles. As shown in Figure 8, no fluorescence signal was observed when the cells were exposed with NPs, which results from the fact that control NPs effectively inhibited the drug release outside medium as well after internalization. There was little or no fluorescence observed in the cells. Interestingly, the effective cell uptake and remarkable increase in PpIX was observed when SCC7 cells were exposed with PpIX-pH-NPs. The main target of photodynamic drugs is cytoplasm of the cellular organelles. From the cellular uptake images, it is clear that PpIX-pH-NPs can release the PpIX specifically to the cytoplasm of cancer cells, as observed by clear red spots. In vitro release test and cellular uptake studies showed that PpIX-pH-NPs could reduce the release at physiological

### 3.6. In vitro phototoxicity of nanoparticles

**Figure 9:**
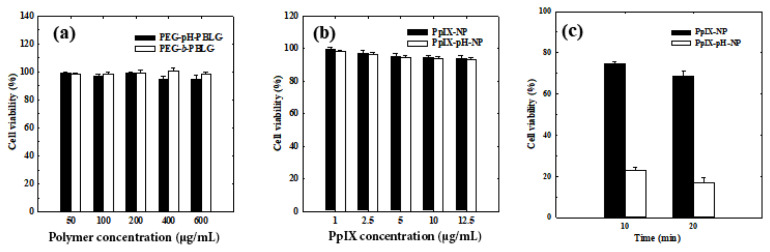
(a) In vitro cell viability of SCC7 cells after incubating with different concentrations of PEG-pH-PBLG and PEG-*b*-PBLG copolymers. (b) In vitro cell viability of SCC7 cells after incubating with different concentration of PpIX-pH-NPs and PpIX-NPs without laser irradiation. (c) In vitro cell viability of SCC7 cells treated with PpIX-pH-NPs and PpIX-NPs under laser irradiation for 5 and 10 min.

In general, PpIX photosensitizer does not produce any toxicity in the absence of light. Upon exposure of near infrared light with appropriate wavelength, PpIX generates singlet oxygen and kill cancer cells. Before the phototoxicity studies, the biocompatibility of nanoparticles was examined to confirm the nontoxic property of nanoparticles. As expected, both nanoparticles showed nontoxic to SCC7 cancer cells at high concentration tested (600 μg/mL), suggesting biocompatibility of nanoparticles (Figure 9a).

To observe the phototoxicity, cells were treated with PpIX-loaded nanoparticles and their toxicity to SCC7 cells were observed with and without exposure of irradiation with a 671 nm He-Ne laser. As anticipated, PpIX-NPs showed negligible toxicity to SCC7 cells under dark condition (without laser irradiation) as well as laser irradiation condition (Figures 9b and 9c). Based on the release test, it is clear that PpIX release from the control nanoparticles is significantly low under both pH conditions, and such a low concentration of PpIX may not generate enough singlet oxygen to induce cancer cell death. In general, upon suitable laser exposure, photosensitizers including PpIX generate singlet oxygen and induce apoptosis due to the high reactivity of singlet oxygen (^1^O_2_). The highly reactive ^1^O_2_ can react with lipids, proteins, and nucleic acids, which eventually induce cellular damages (Liang et al., 2020; Maharjan and Bhattarai 2022). Interestingly, when irradiated with 671 nm He-Ne laser, the cells in the PpIX-pH-NPs treatment group were effectively killed. At the same time, PpIX-pH-NPs did not induce any toxicity in dark conditions, indicating that PpIX-pH-NPs control the drug release and specifically induce toxicity to cancer cells. This PpIX-pH-NPs could also perform better in in vivo as reported by Lee et al., in which release of PpIX from polymeric conjugates in a sustained and tumor specific manner induced enhanced phototoxicity in lung-cancer-bearing mice (Lee et al., 2009) (Lee et al., 2011).

## 4. Conclusion

In conclusion, we developed pH-NPs that exhibit disassembly at the tumor acid pH condition for the site-specific delivery of photosensitizer to cancer cells. In aqueous solutions, the pH-NPs effectively self-assembled to form stable nanoparticles with a mean diameter of 150 nm. The pH-NPs effectively load PpIX in the hydrophobic core with no leakage at the physiological condition. At the acidic pH condition, the pH-NPs are disassembled and trigger the release of PpIX. From in vitro toxicity test, it is found that PpIX-pH-NPs exhibit enhanced toxicity upon NIR irradiation, which are nontoxic under dark condition. Such an on-off system with site-specific release of PpIX from nanoparticles can reduce unwanted toxicity and enhance therapeutic efficacy. These results suggest that pH-NPs have great potential in PDT-based cancer treatment.

## Figures and Tables

**Table:** Physicochemical characteristics of the pH-responsive block copolymers.

Sample name	Feed ratio^a^	DP^b^	Size (nm)^c^	Mn^d^
PEG-pH-PBLG	15	12.12	130	7654
PEG-*b*-PBLG	15	13.06	145	7860

a Molar feed ratio of benzylglutamate NCA to PEG-pH-NH_2_.

b Degree of polymerization by ^1^H NMR spectra.

c Mean diameter measured calculated using the particle analyzer.

d Number-average molecular weight calculated using ^1^H NMR spectra.
